# The Gamma and Neutron Sensor System for Rapid Dose Rate Mapping in the CLEANDEM Project

**DOI:** 10.3390/s23094210

**Published:** 2023-04-23

**Authors:** Fabio Rossi, Luigi Cosentino, Fabio Longhitano, Saverio Minutoli, Paolo Musico, Mikhail Osipenko, Gaetano Elio Poma, Marco Ripani, Paolo Finocchiaro

**Affiliations:** 1 INFN Sezione di Genova, 16146 Genova, Italy; fabio.rossi@ge.infn.it (F.R.); saverio.minutoli@ge.infn.it (S.M.); paolo.musico@ge.infn.it (P.M.); mikhail.osipenko@ge.infn.it (M.O.); marco.ripani@ge.infn.it (M.R.); 2 INFN Laboratori Nazionali del Sud, 95123 Catania, Italy; cosentino@lns.infn.it (L.C.); elio.poma@lns.infn.it (G.E.P.); 3 INFN Sezione di Catania, 95123 Catania, Italy; fabio.longhitano@ct.infn.it

**Keywords:** gamma detector, neutron detector, nuclear decommissioning, nuclear accident remediation, robotic radiological inspection

## Abstract

The decommissioning of nuclear installations, as well as the possible necessary accident remediations, requires the physical presence of human operators in potentially radiologically hostile environments. The number of active nuclear reactors worldwide is greater than 400, and most of them are 40 to 50 years old, thus implying that soon they will have to be dismantled. In the framework of the H2020 CLEANDEM project, a small robotic vehicle is being developed that is equipped with a series of different sensors for areas that are significantly contaminated by radiation. In this work, we describe the MiniRadMeter system, a compact low-cost sensor capable of being used to perform quick gamma and neutron radiation field mapping of environments prior to the possible start of human operations. The miniature gamma sensor is a 1 cm^3^ scintillator counter with moderate spectroscopic features read out by means of a 6 × 6 mm^2^ SiPM, whereas neutrons are detected by means of a silicon diode coupled to a layer of ^6^LiF and placed inside a 6 × 6 × 6 cm^3^ polyethylene box. The front-end and data acquisition electronics were developed based on a Raspberry Pi4 microcomputer. In this paper, the system performance and the preliminary test results are described.

## 1. Introduction

**Figure 1:**
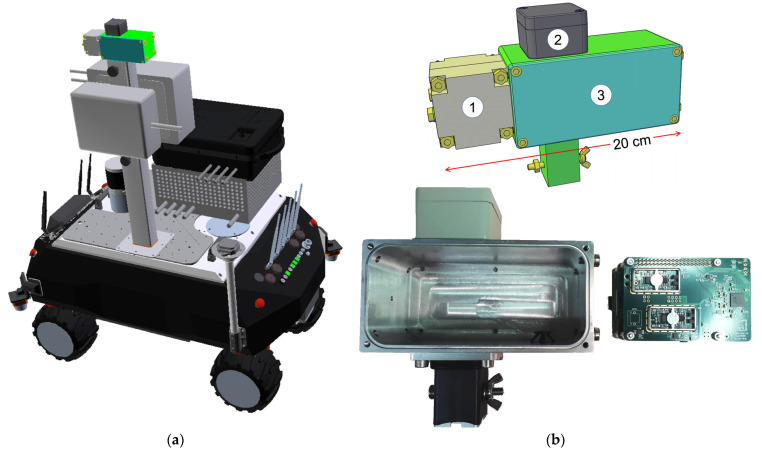
(**a**) A 3D sketch of the CLEANDEM UGV under development. (**b**) The MiniRadMeter device hosting: the neutron moderator and detector (1), the gamma detector (2), the front-end electronics, and the data acquisition microcomputer (3). In the bottom picture the neutron moderator was not yet installed.

The worldwide number of active nuclear reactors is greater than 400, and most of them are 40 to 50 years old, which implies that soon they will have to be dismantled. The decommissioning of nuclear installations, as well as possible accident remediations, generally requires the physical presence of human operators in potentially radiological hostile environments and, therefore, presents the consequent risks. Recent technologies promise the development of new unmanned systems with much safer features, based on the simple assumption “first inspect, then decide”; because of the progress of miniature mechanical and electronic systems, one can imagine a remotely operated robotic inspection vehicle capable of a preliminary reconnaissance and mapping of the site undergoing decommissioning. Similarly to this, the CLEANDEM project (Cyber Physical Equipment for Unmanned Nuclear Decommissioning Measurements) was recently funded in the framework of the EU Horizon 2020 plan [1]. The UGV (Unmanned Ground Vehicle) being developed, as shown in Figure 1a, has an onboard computer with a wireless connection to its remote console, and is connected to several devices and sensors installed onto its service platform and on a suitable robotic arm. A wide range of sensors currently under development can be installed on the UGV, and these sensors can be added depending on the outcome of a preliminary reconnaissance mission and the consequent type of operations or further investigations to be performed. The full description of the UGV and of all its sensors will be the subject of forthcoming papers.

As is well known, the main types of radioactivity are alpha, beta, gamma, neutron, and fission. On the one hand, alpha particles and fission fragments are stopped immediately by aluminum foils of about 25 and 15 µm thickness, respectively, with beta electrons traveling a little further but still being stopped by a 4 mm aluminum sheet. On the other hand, neutrons and gamma rays are much more penetrating and may require tens of centimeters of lead, concrete, and/or water to be stopped. This implies that they are much easier to detect; detecting alpha and beta radiation or fission fragments in air requires placing delicate detectors with very thin entrance windows closely to the emitting objects, whereas the detection of gamma rays and neutrons can be performed from a distance (e.g., 1 m) with rugged detectors. This is why preliminary radiological reconnaissance should primarily focus on gammas and neutrons, considering that most types of radioactive decay usually involve the emission of gamma radiation, while neutrons indicate the presence of alpha-emitting actinides. 

Detection for dosimetric purposes is typically based on passive detectors, and would not be suitable for the purpose of this work [2]. Active detectors for gamma rays are mainly based on scintillators, a rugged consolidated technology, or on more sophisticated solid-state detectors, such as Germanium or CdTe [3]. The detectors that are mainly used for neutrons are the ^3^He proportional counters, which are expensive and require a high voltage and, typically, a moderator in the form of a Bonner sphere [4]. The reduced availability, and the consequent price increase in ^3^He over the last 15 years has led to the development of several alternative technologies for neutron detection, such as the SiLiF solid-state detectors.

In this paper, we are going to describe a device named MiniRadMeter, as shown in Figure 1b, which will be permanently installed on the topmost position of the UGV, with the aim of performing fast gamma and neutron dose rate monitoring. Even though the current version this device will be fixed on the UGV, in future applications it could be easily operated via battery and deployed extensively across an accident site, due to its compactness and low cost. 

In Section 2, the main detection techniques and components of the MiniRadMeter system are described, namely the gamma and the neutron detectors and their characterization with typical nuclear electronic modules. Section 3 is dedicated to the description of the newly developed compact electronic front-end and data acquisition system. Then, in Section 4, the preliminary results obtained with the complete MiniRadMeter system are shown, in view of a forthcoming demonstration in a real environment. 

## 2. Materials and Methods

### 2.1. Gamma Ray Detection

Reasonably quick dose rate monitoring and mapping of an unknown location depends on the foreseen dose rates and on the geometry and type of the possible environments to be investigated. Its duration should not exceed the duration of the UGV batteries (currently 2–3 h) and should detect relevant radiological activity, leaving the possible finer investigation to more specialized devices. In order to detect gamma rays, it was decided to employ a scintillating crystal, which is an effective and consolidated technology [5,6]. The chosen material was CsI(Tl), due to the fact that it is well known for its high-quality detection properties in terms of detection efficiency (i.e., high density), light yield, and energy resolution at a reasonable cost (Table 1).

**Figure 2:**
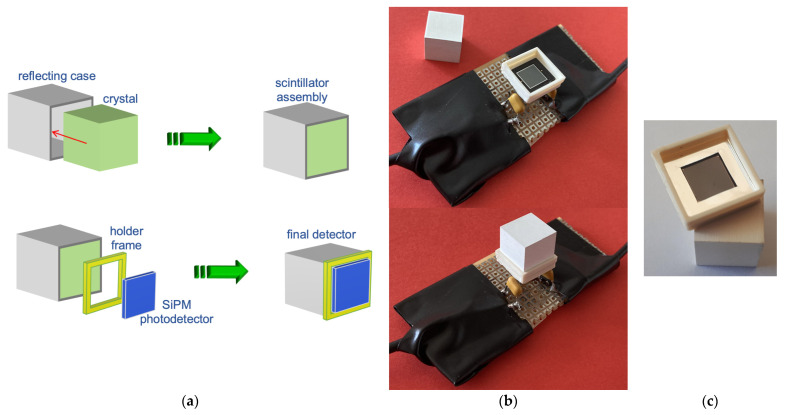
(**a**) A 3D sketch of the gamma detector assembly. (**b**) The gamma detector preliminary prototype components, the SiPM is visible inside the frame (top). The detector assembled (bottom). (**c**) An aluminized reflecting frame that covers the black insensitive part of the SiPM to improve the light collection.

The chosen geometry was a small cube of 1 × 1 × 1 cm^3^ in size encased in a five-face box of white reflective epoxy resin that was 0.2 mm thick, produced by Hilger Crystals, Margate, UK. On the free face, a 3D-printed frame was installed to accommodate a SiPM photodetector, the MicroFC-60035-SMT [7] produced by SensL, Cork, Ireland (now OnSemi, Phoenix, AZ, USA), with 6 × 6 mm^2^ active area and 18,980 microcells, optically coupled to the crystal by means of optical grease. Figure 2a sketches the structure of such a setup, while, in Figure 2b, the real detector is shown, before and after its assembling, installed on a test PCB. In Figure 2c, the aluminized reflective frame that covers the black insensitive part of the SiPM to improve the light collection is shown. 

The expected performance of the detector was calculated with respect to several features. Additional details about the features of other similar detectors can be found in [8,9,10,11,12,13,14,15,16,17]. First of all, the detected photon statistics versus the produced light was investigated. An impinging gamma ray of 2 MeV releasing all its energy into the crystal was considered. The scintillation light yield was assumed as the commonly accepted 60,000 photons/MeV and its exponential decay was constant at 960 ns. As for the SiPM, the reported photon detection efficiency at the 550 nm average wavelength of the CsI(Tl) light is between 15 and 20%, and 18% was assumed [7]. The time evolution of the number of detected photons was calculated as follows. First, the time distribution of the emitted photons was calculated with a time step of 5 ns according to an exponential function with a constant decay of 960 ns and an integral equal to the total number of photons. The SiPM datasheet reports a microcell recharge time of 95 ns [7], and 100 ns was assumed. At each time step, the number of fired cells was calculated by multiplying the number of photons by the detection efficiency, taking into account the multiple hit probability and the available number of active cells. The fired cells were removed from the total number of available cells for the following 100 ns, then declared active again. It was found that the number of fired cells per time step is small and the difference with respect to the ideal case with an infinite number of cells and zero recharge time is negligible. Indeed, in the case of a 2 MeV energy deposition, the calculated number of fired cells in the first time step is 111.8, with the ideal being 112, with 18,980 initially available cells. The maximum decrease in available cells, down to 16,852, occurs after 100 ns and still gives rise to an expected loss of only 0.3 photons (101.2 versus 101.5) because the light intensity, i.e., number of emitted photons, decreases exponentially with time.

**Figure 3:**
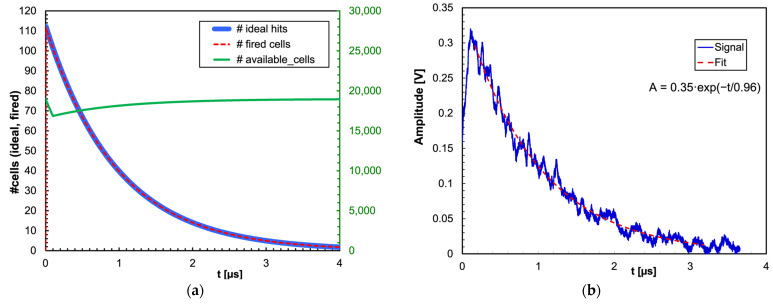
(**a**) The number of expected ideal photon hits (*dPh/dt·*∆*t*) as a function of time for a 2 MeV gamma energy deposition is basically indistinguishable from the calculated number of real cell hits. The number of available cells as a function of time is also plotted (in green, right-hand axis). (**b**) A real signal, after a linear amplification, along with an exponential fit that (not surprisingly) produced the expected 960 ns decay constant. The signal fluctuations are to be ascribed to photon statistics due to the small number of expected hits.

As a conclusion, due to the small number of impinging photons per unit of time compared to the number of SiPM cells, and due to their short recharge time, the effect of multiple hit and cell recharging is negligible, as can be seen in Figure 3a, where the number of expected ideal photon hits (*dPh/dt·*∆*t*) is basically indistinguishable from the calculated number of real cell hits. This means that the detector does not suffer from any non-linearity because of the finite number of SiPM microcells, nor because of microcells being unavailable during their recharge after being fired. In Figure 3b, a real signal is shown, after a linear amplification, along with an exponential fit that (not surprisingly) produced the expected 960 ns decay constant of CsI(Tl). Remarkably, the signal fluctuations are to be ascribed to photon statistics due to the small number of expected hits.

**Figure 4:**
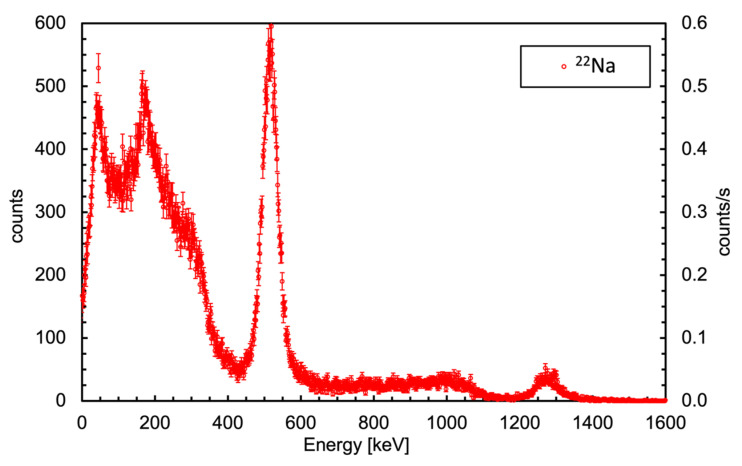
Gamma spectrum measured using a ^22^Na source. The energy calibration was performed by means of the 511 and 1274 keV peaks. The linearity is confirmed by the Compton backscattering peak falling exactly at 170 keV as expected. The energy resolution measured at 511 keV is 9% FWHM.

The detector was then connected to a Charge-to-Digital Converter, namely the DT5740 [18] produced by CAEN, Viareggio, Italy, and exposed to a ^22^Na radioactive source which decays via beta+ and gamma, giving rise to two annihilation gamma rays of 511 keV and one of 1274 keV. The resulting spectrum, calibrated in energy by means of the 511 and 1274 keV peaks, is reported in Figure 4. The linearity was confirmed by the Compton backscattering peak falling at 170 keV as expected, and the energy resolution measured at 511 keV was 9% FWHM.

**Figure 5:**
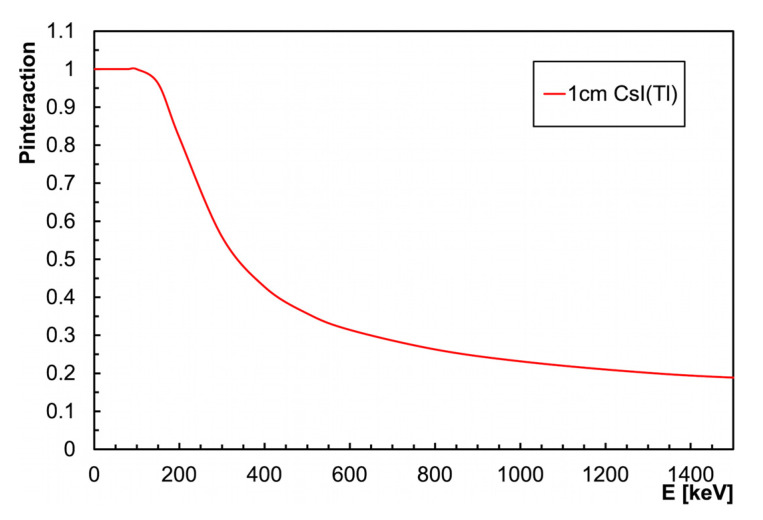
Interaction probability in 1 cm thick CsI(Tl) as a function of the gamma energy.

The 1 cm^3^ CsI(Tl) crystal coupled to a SiPM, with an overall weight of about 5 g, provides linearity and energy resolution in line with the expectation from existing literature [8,9,10,11,16,17]. It can, thus, be used preliminarily in simple counting mode then, whenever deemed necessary, it can also be used in a spectroscopic mode for a coarse identification of the gamma emitting radionuclides. The dosimeters are usually calibrated based on the 662 keV gamma radiation from ^137^Cs, for which the equivalent dose rate at 1 m is 0.1 µSv/h/MBq. A simple geometrical calculation shows that 1 MBq at 1 m distance produces a flux of about 8 gamma/cm^2^/s. The interaction probability of such a gamma ray with a 1 cm thick CsI(Tl) crystal is about 30% (Figure 5, data from [19]); therefore, the expected counting rate, also verified experimentally, is about 2.5 cps/MBq (cps: counts per second) or, in equivalent dose rate, 25 cps/(µSv/h). 

**Figure 6:**
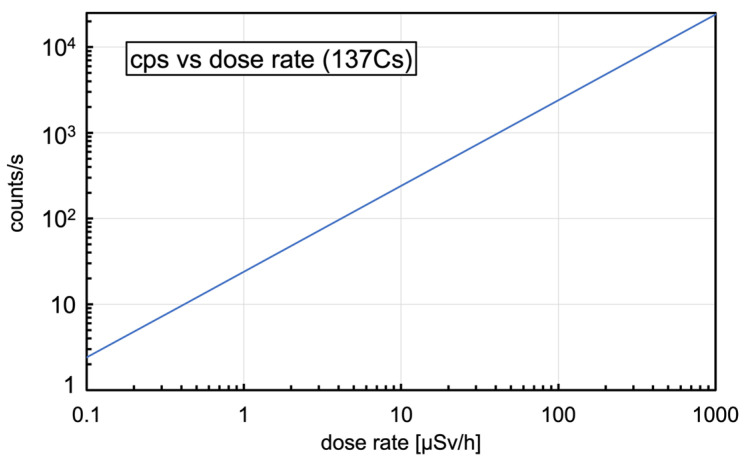
The estimated counting rate as a function of dose rate for ^137^Cs gamma radiation.

**Figure 7:**
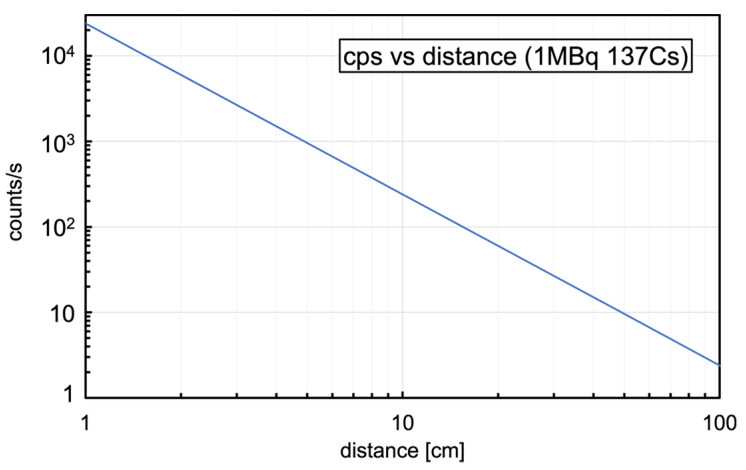
The estimated counting rate as a function of the distance from a point-like 1 MBq ^137^Cs source.

**Figure 8:**
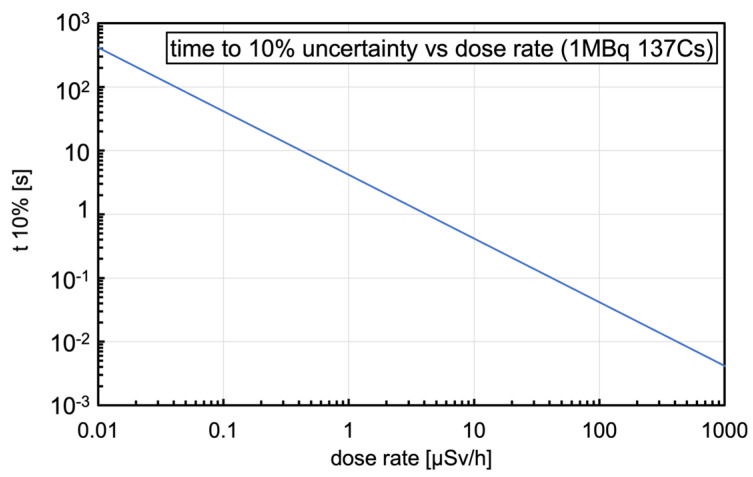
The time to 10% uncertainty (i.e., time required for 100 counts to be registered) as a function of dose rate for ^137^Cs gamma radiation.

These numbers make possible a quick estimate of the expected performance as a dosimeter when operating in counting mode, and three simple reference plots useful for quick lookup were built; Figure 6 shows the counting rate as a function of the dose rate, Figure 7 shows the counting rate as a function of the distance from a point-like 1 MBq ^137^Cs source, and Figure 8 shows the time to 10% uncertainty (i.e., time required for 100 counts to be registered) as a function of the dose rate.

### 2.2. Neutron Detection

**Figure 9:**
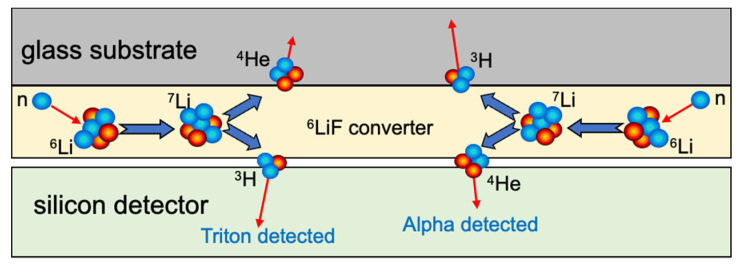
Simple sketch of the operating principle of the employed SiLiF technique.

As a neutron is a neutral particle, its detection has to be based on a so-called neutron conversion, a nuclear reaction where the neutron interaction produces other particles easily detectable by means of their electromagnetic interaction with matter. Building a small low-cost detector for quick neutron rate monitoring was not a simple task [2,4], and a trade-off between cost, detection efficiency, and overall detector size was needed, as required by the project guidelines and by the operational features of the UGV and the other systems it will host. In addition, an optimum gamma/neutron discrimination was required, along with a sufficient robustness and compactness that would enable installation onboard a UGV. Therefore, in order to detect neutrons it was decided to employ a miniature solid state device based on the SiLiF technique [20,21,22,23,24,25]; a layer of ^6^LiF is deposited onto a suitable substrate and is then coupled to a silicon diode which detects the tritons and/or alphas produced by the neutron capture on ^6^Li according to the following reaction,

$$n+{}^{6}\mathrm{Li}\to {}^{3}H+{}^{4}\mathrm{He}$$

which has a thermal cross section of 940 b, with the kinetic energy of the two back-to-back emitted particles being 2.73 and 2.05 MeV, respectively. A simple sketch of the operating principle of the employed SiLiF technique is shown in Figure 9. Indeed, there are other nuclear species with large thermal neutron cross-sections, but they were deemed unsuitable for the current application, as described in Table 2.

**Figure 10:**
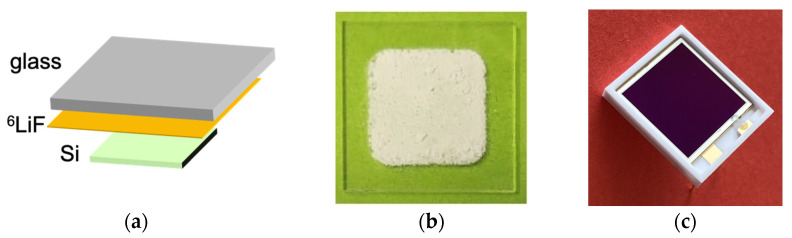
(**a**) A 3D sketch of the neutron detector assembly. (**b**) The ^6^LiF converter chemically deposited onto a glass substrate. (**c**) The S3590-09 PIN photodiode.

According to the long standing SiLiF technique [20,21,22,23,24,25], a ^6^LiF layer of 4300 µg/cm^2^ areal density (≈17 µm thickness at nominal density), enriched at 95% in ^6^Li, was deposited onto a glass substrate by means of a reasonably simple chemical procedure [34]. As for the silicon diode, an off-the-shelf PIN photodiode of 1 cm^2^ area and 300 µm thickness was chosen, namely the unsealed S3590-09 [35] produced by Hamamatsu Photonics, Hamamatsu, Japan, costing about EUR 100, which has no transparent resin window in front, so that the particles can reach the depleted junction region. The thermal neutron efficiency of such a setup is between 2.5% and 3.5%, depending on the adopted threshold on the deposited energy which determines the gamma/neutron discrimination performance [21]. Figure 10 shows a sketch of the detector assembly, a picture of the converter and one of the silicon diode.

**Figure 11:**
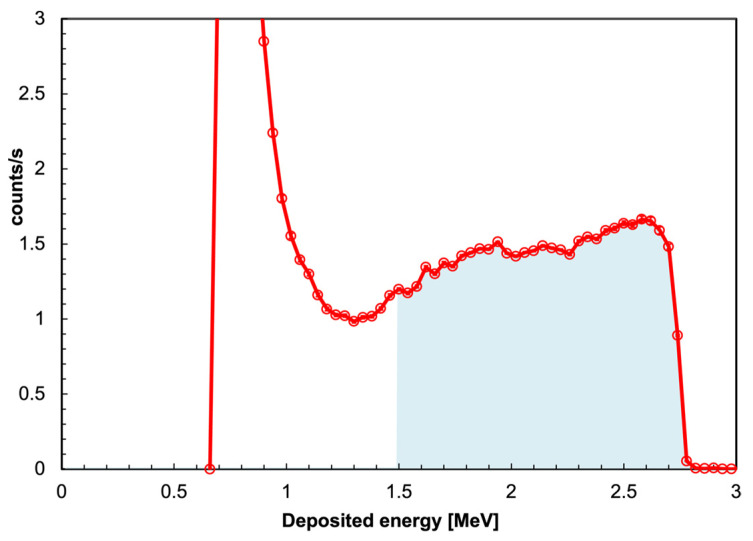
The typical whale-shaped SiLiF deposited energy spectrum, with the neutron region above the 1.5 MeV threshold highlighted in blue.

As is well known, the neutron capture cross-section is high at thermal energy and decreases as 1/*v* rises to a few hundred keV, which implies that, in order to improve the response at higher energy, one has to slow down the incoming neutrons by means of a suitable moderator. The thermal neutron intrinsic detection efficiency of the detector, if enforcing a selection threshold at 1.5 MeV on the deposited energy, is about 2.5% with a gamma/neutron rejection of the order of 10^−10^ [21,36]. Figure 11 shows the typical whale-shaped deposited energy spectrum, with the highlighted threshold at 1.5 MeV, above which all the counts are considered as produced by neutrons. Despite the low detection efficiency, the superior gamma/neutron discrimination makes it possible to state the presence of actinides whenever counts are reported by this detector. 

**Figure 12:**
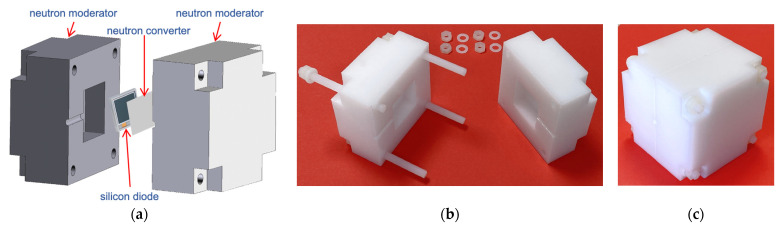
The 6 × 6 × 6 cm^3^ polyethylene moderator, a suitable groove for the connection cable is visible. (**a**) A 3D assembly sketch. (**b**) The real moderator, open. (**c**) The real moderator, closed.

**Figure 13:**
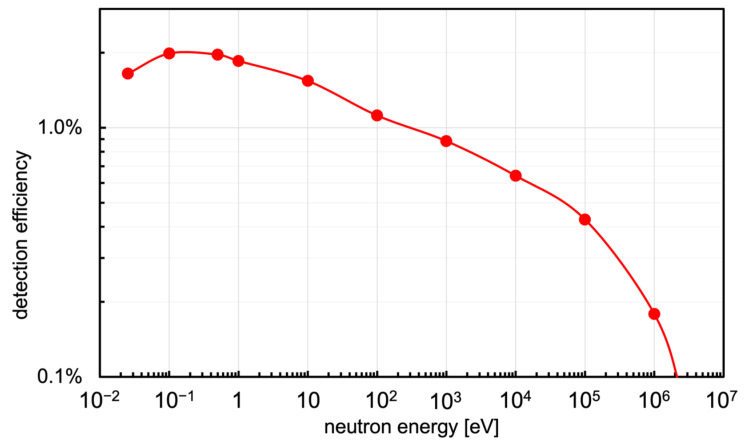
Detection efficiency as a function of the neutron energy for the SiLiF detector placed inside the moderator.

In order to moderate the incoming neutrons a polyethylene moderator 6 × 6 × 6 cm^3^ was designed and built (Figure 12). Its moderation performance was simulated with the GEANT4 code [37], by calculating the detection efficiency of the SiLiF detector inside it as a fraction of the detected neutron flux when perpendicularly irradiating one 6 × 6 cm^2^ face with monoenergetic neutrons. This was repeated for several neutron energies and the resulting plot is shown in Figure 13. Should a better efficiency be needed beyond 1 MeV, a bigger moderator can be employed, e.g., 10 × 10 × 10 cm^3^ [36], with the polyethylene weight increasing from ≈200 g to ≈900 g. In case one were only interested in thermal neutrons, the moderator can be removed leaving the bare SiLiF detector in operation. 

The detection efficiency can be increased, if required, by increasing the number of SiLiF detectors inside the moderator. Indeed, due to the low capacitance of the depleted silicon diode (≈40 pF) up to 10 or more sensors can be connected in parallel and handled by a single readout channel. However, the cost of ten diodes would be equivalent to one double sided 3 × 3 cm^2^ silicon diode, which offers the possibility of doubling its detection efficiency by placing a converter on each side in a sandwich configuration. 

**Figure 14:**
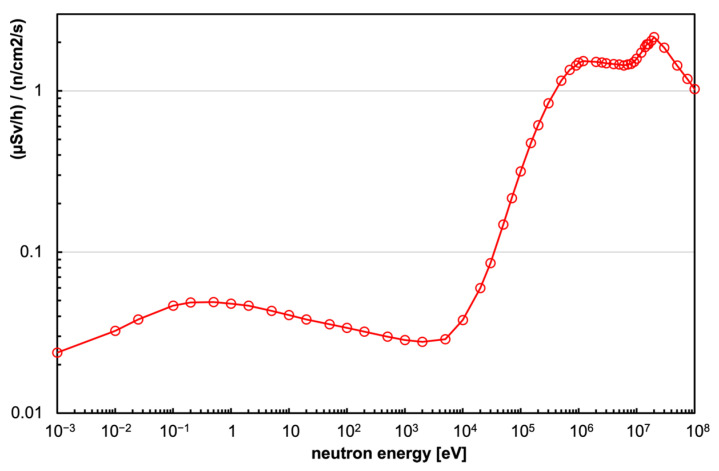
The ratio between the equivalent dose rate (in µSv/h) and the corresponding neutron flux (in neutrons/cm^2^/s) as a function of neutron energy [38].

**Figure 15:**
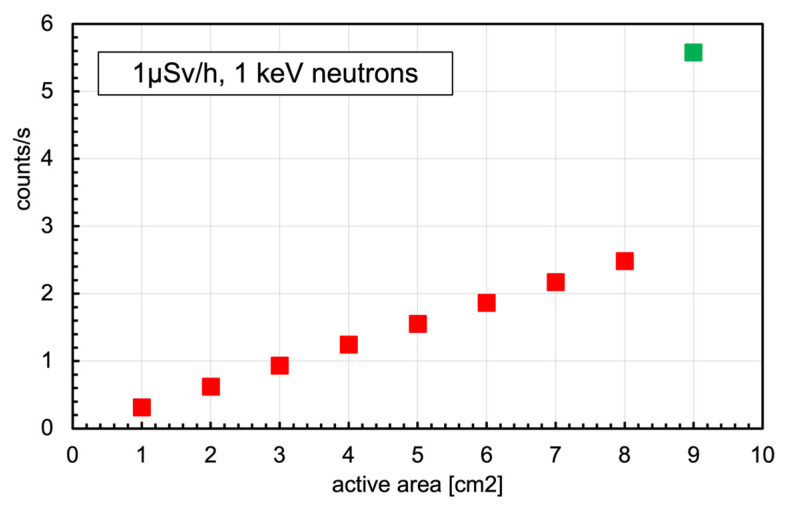
Simple reference plot showing the expected counting rate in a 1 keV neutron field producing 1 µSv/h dose rate as a function of the number of SiLiF units installed. The last point, in green at 9 cm^2^, refers to the case of a 3 × 3 cm^2^ double-sided silicon diode.

**Figure 16:**
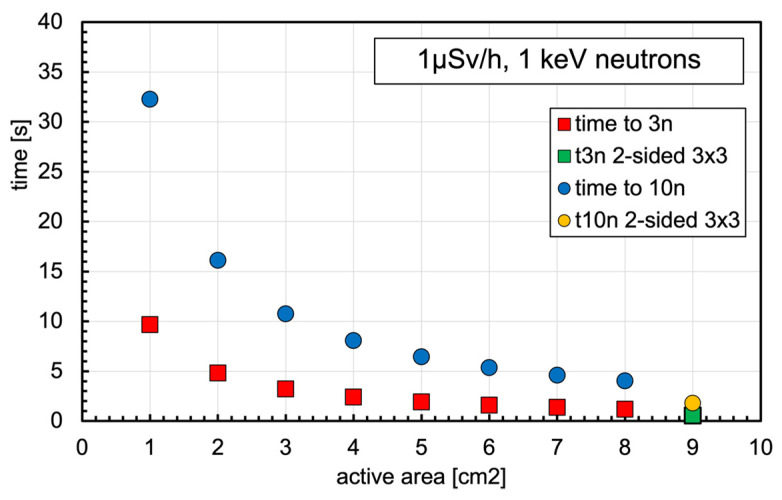
Average expected time to report 3 (red squares) or 10 (blue circles) neutron counts in the detector, with the same assumptions of Figure 15. The green square and the orange circle at 9 cm^2^ refer to the case of a 3 × 3 cm^2^ double-sided silicon diode.

As is known, the equivalent dose for neutrons strongly depends on the neutron energy, being low at thermal energy and jumping up, as shown in Figure 14, where the ratio between the equivalent dose rate (in µSv/h) and the corresponding neutron flux (in neutrons/cm^2^/s) is reported as a function of neutron energy [38]. Neutrons of 1 keV were selected as a reference case, driven by a simplistic assumption that in an internal environment, with the possible presence of a neutron field, a good fraction of the flux is due to neutrons bouncing back and forth, therefore partially moderated by the present material (floor, walls, metallic structures, etc.). In Figure 15, a simple reference plot shows the expected counting rate in a 1 keV neutron field producing 1 µSv/h dose rate as a function of the number of SiLiF units installed. The last point, at 9 cm^2^, refers to the case of a 3 × 3 cm^2^ double sided silicon diode. With the same assumptions, the plot of Figure 16 shows the average expected time to report counting 3 or 10 neutrons in the detector. 

## 3. Electronics and Data Acquisition

The data acquisition system consists of two analog front-ends, one for each sensor, a single ADC with two differential input channels and a Raspberry Pi4 microcomputer board [39] that collects, elaborates, and saves data and/or sends them to the back-end software. Employing a microcomputer instead of faster FPGA technology was preferred in this phase because of the easier programming and portability, even though a future migration to an FPGA-based system is not excluded. The back-end user interface runs on a standard PC and it is programmed in Python. During the measurement mission, it will build and show the detector spectra and counting rates for both sensors, also showing, on request, the signals in the time domain or after their shaping. By means of the graphical user interface, it is also possible to handle all the acquisition and data storage parameters, as well as select the kind of trigger and data collection mode that has to be employed. The connection between the Raspberry and the back-end can be performed either by means of an ethernet connection to the UGV, which then takes care of the Wi-Fi routing, or directly via the onboard Wi-Fi interface.

**Figure 17:**
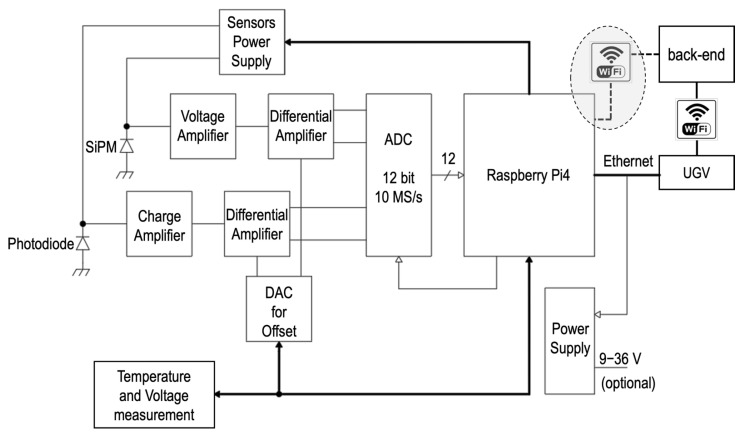
Simplified block diagram of the front-end and data acquisition system.

Figure 17 shows a simplified block diagram of the front-end and data acquisition system that includes the analog and the digital part. The analog part consists of operational amplifiers, one for each sensor. The digital part is identical for the two sensors thanks to the double input ADC that features a sampling frequency up to 10 MS/s and a 12-bit parallel output directly connected to the Raspberry GPIO (General Purpose Input Output). The Raspberry provides all the ADC control signals, including the Clock reference. On the board there are two step-up DC–DC converters to supply bias voltage for sensors, controllable via SPI (Serial Peripheral Interface) in order to adjust the right polarization voltage. In addition, with an I^2^C (Inter Integrated Circuit) temperature sensor it is also possible to compensate for the dependence of the breakdown voltage of SiPM on temperature. The I^2^C bus is also used to monitor and feedback the bias voltages by means of two slow onboard 8-bit ADCs. The last element is a dedicated PCB developed to supply the overall system voltage. As the system will be installed on a battery powered UGV, the data acquisition system has been designed to have very low power consumption. It requires about 2.5 W in idle mode and 6 W when in operation, which can be taken directly via PoE (Power Over Ethernet), or via a general power supply provided by the UGV (12 or 24 V) or via USB 3.0. However, the PoE solution has been preferred so far because it minimizes cabling on the UGV.

### 3.1. Analog Front-End

**Figure 18:**
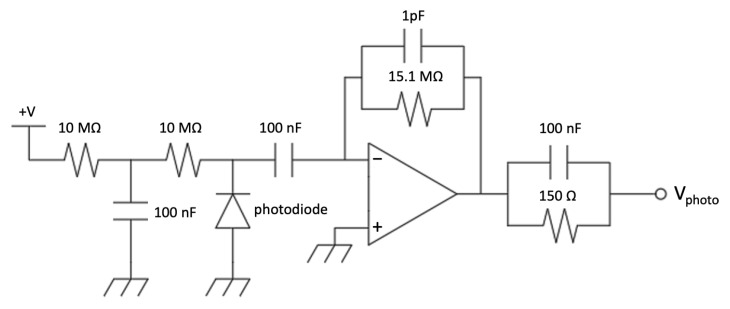
The charge integrating amplifier (first stage) of the silicon diode front-end.

**Figure 19:**
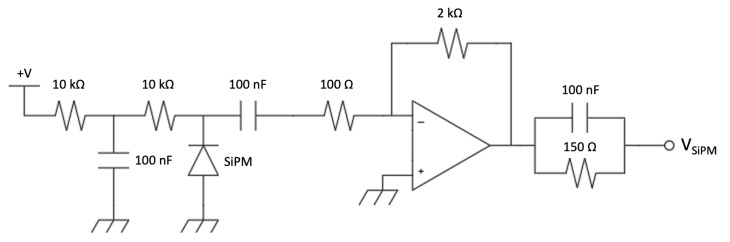
The voltage amplifier (first stage) of the SiPM front-end.

The signal from each sensor is picked up on the cathode and by capacitive coupling it is amplified by means of an operational amplifier stage in inverting configuration. A charge-integrating amplifier was used for the silicon diode (Figure 18) and a voltage amplifier for the SiPM (Figure 19). The charge amplifier features a high-speed operational amplifier to ensure a proper charge integration of the fast silicon diode pulse signals. The gain was set to 1 V/pC and the decay time of 15 µs was chosen as a trade-off between the number of samples that the ADC can convert in each pulse and the expected signal rate to minimize the occurrence of pile-up. The voltage amplifier features the same operational amplifier and circuit topology to simplify the PCB and make it more flexible, but it was assembled without the feedback capacitor and with a different resistor ratio to obtain a voltage gain of 20. 

**Figure 20:**
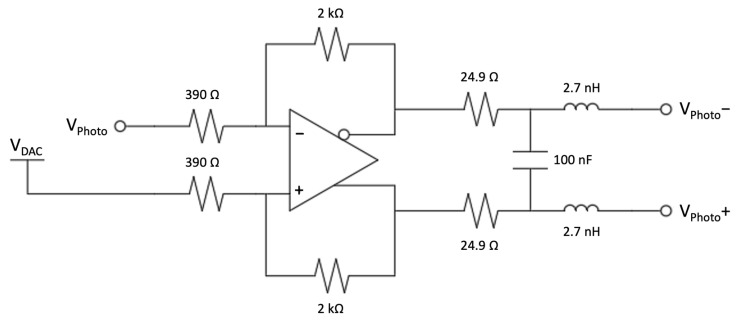
The second amplifying stage of the silicon diode front-end.

**Figure 21:**
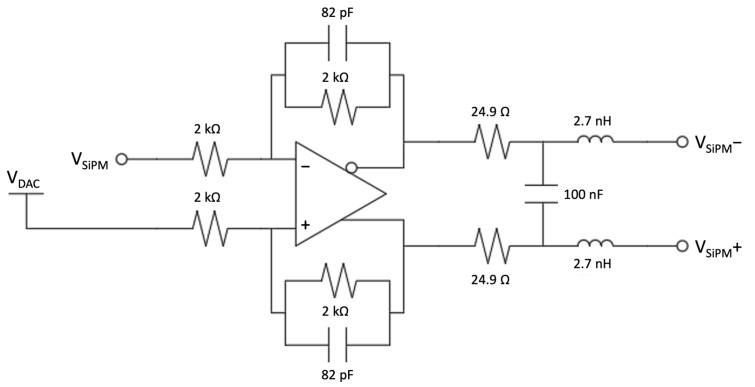
The second amplifying stage of the SiPM front-end.

The amplifier is followed by a pole-zero cancellation RC network and another voltage amplifier with a differential output. The differential output is required to interface the front-end with the ADC and to add an offset, generated with a software controlled DAC, in order to use the full ADC voltage range and to compensate, by means of a calibration routine, the possible offset introduced by the tolerance of components (Figure 20 and Figure 21). This stage is useful on the one hand to increase the silicon diode gain and on the other hand to filter the SiPM signals. In order to match the ADC range, the silicon diode requires a total gain of 5 V/pC, not easily achievable with only one stage due to stability issues and limits on the values of the components. The anti-aliasing filter for the SiPM, with a cut-off frequency of 2.2 MHz was not enough to shape the signal; therefore, an active filter with a cut-off frequency of 1 MHz was introduced in the differential stage. In this way, the signal is slowed down in order to collect a sufficient number of samples at 10 MS/s for the measurement.

### 3.2. Data Acquisition

**Figure 22:**
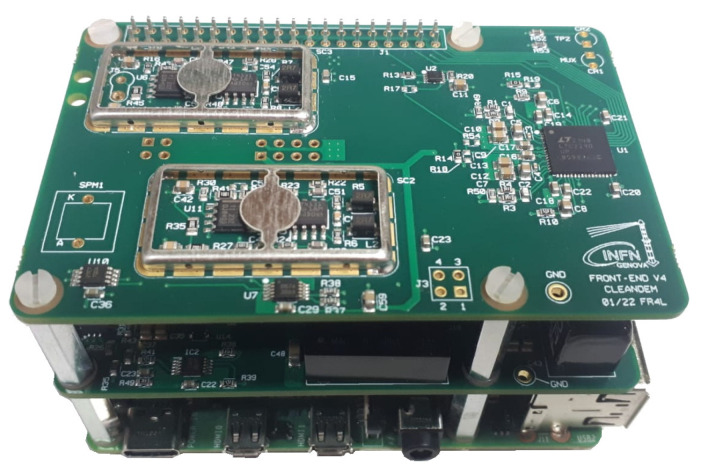
The Raspberry Pi4 board assembled with the front-end electronics and the ADC.

The data acquisition is carried out by a Raspberry Pi4 equipped with a 4-core Arm Cortex A-72 processor (Figure 22). The operating system (OS) is a standard Raspbian distribution (Debian version 11) which is not real-time, so its time response is not deterministic. On the other hand, the acquisition of sensor data must be deterministic with each voltage sample equally spaced in time, and the ADC operation requires a stable clock with constant duty cycle. 

**Figure 23:**
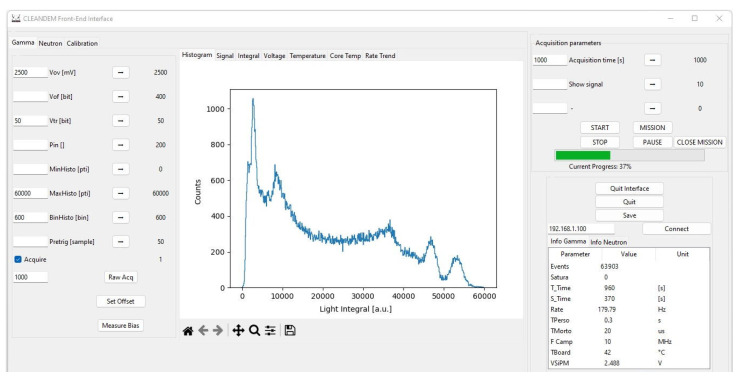
A snapshot of the developed Graphical User Interface (GUI).

In order to be able to measure with this system, some low-level code in C language was developed to directly drive the microprocessor peripherals, and thus bypassing the OS layer. A dedicated memory handling function was written to overcome limitations arising from the different addressing space used by the running code (Virtual Memory), the peripheral registers (Physical Memory), and the DMA (Bus Address Space) which must not be cached and, thus, has to be allocated in well-defined physical positions in the addressing space. In order to optimize the data acquisition operations, the four cores available in the processor were charged with specific tasks. A detailed description of the software structure and of the implemented algorithms will be the subject of a forthcoming paper. A snapshot of the developed GUI (Graphical User Interface) is shown in Figure 23. 

The incoming signal from each second stage amplifier is continuously sampled by the ADC at 10 MS/s and the sequence of samples is stored into memory blocks. The data recorded in a memory block are scanned searching for valid signals by means of a software leading-edge trigger; when a number of consecutive samples are found above the threshold a valid pulse is declared and a shaping routine starts. The code to handle the neutron and the gamma sensor data are the same except for the shaping algorithm. 

**Figure 24:**
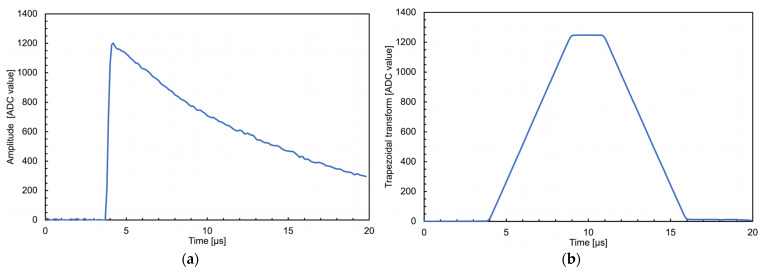
A signal detected by the silicon diode, handled by the front-end and sampled by the Raspberry at 10 Ms/s. (**a**) The signal in the time domain. (**b**) The signal after the trapezoidal shaping.

**Figure 25:**
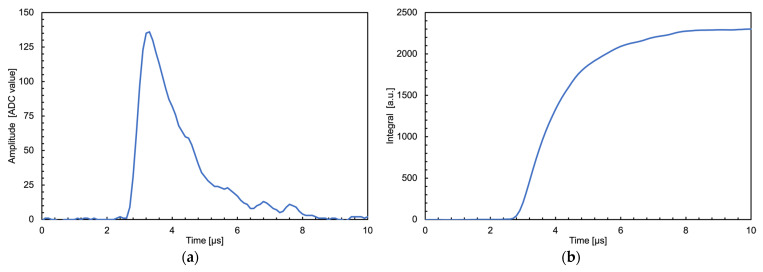
A signal from a ^241^Am gamma ray detected by the CsI(Tl) + SiPM, handled by the front-end and sampled by the Raspberry at 10 Ms/s. (**a**) The signal in the time domain. (**b**) The numerical integral obtained by summing the set of samples above a predefined threshold.

The shaping routines are rather slow in both cases, but they are executed only on the occurrence of valid events that in this application never reach above a few kHz. In the neutron counter case, the routine consists of a recursive trapezoidal transform algorithm [40] and the signal amplitude value, proportional to the energy deposited in the silicon detector, is the mean value of the trapezoid flat top height (Figure 24). In the gamma counter case, a simpler signal integration is computed as the sum of all its sample values (Figure 25). In both cases, the result of each signal shaping is used to compute the counting rate and the histogram. 

## 4. Results

### 4.1. Test of Gamma Ray Detection

**Figure 26:**
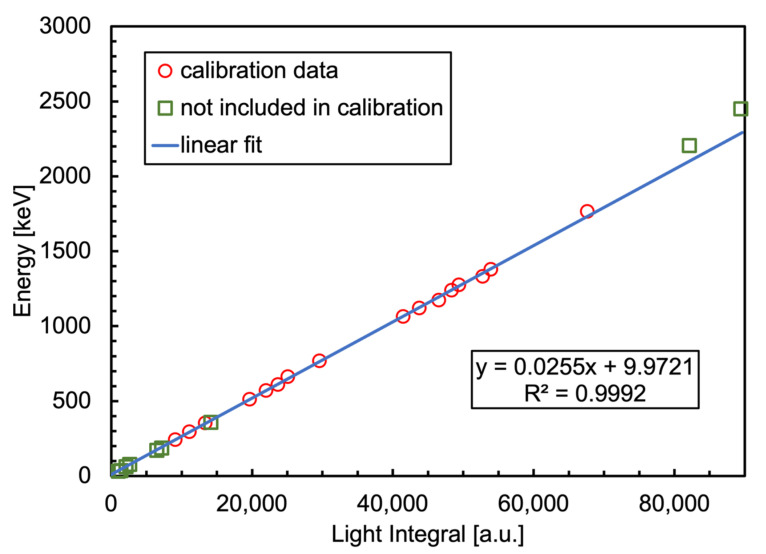
The channel-to-energy calibration plot for the gamma detector. The red circles refer to the gamma energies used for the calibration fit listed in Table 3, the green squares refer to the energies not included in the calibration fit (see the text and Table 3 for details).

The CsI(Tl) crystal was assembled and connected to its front-end electronic board, and was tested with seven laboratory radioactive sources. The main energies of the emitted gamma rays and those that were used for the channel to fit energy calibration of the spectra are listed in Table 3. The low energy peaks below 100 keV were not used for the calibration as they suffered distortion by atomic X-rays from the lead collimator, and other peaks were excluded because of their low statistics. The channel-to-energy calibration plot reported in Figure 26 shows an overall linearity of the detector up to 1800 keV, where it starts to deviate. From the considerations in Section 2.1 and Figure 3, the effect is not to be ascribed to a SiPM saturation, therefore it likely depends on the response of the crystal to higher energy gamma rays, as Ref. [41] seems to indicate. Moreover, the authors of Ref. [41] also pointed out that such an effect is smaller as the integration time is increased, and this is why 20 µs was chosen for the present detector configuration even though it is a programmable parameter that can be easily modified at runtime.

**Figure 27:**
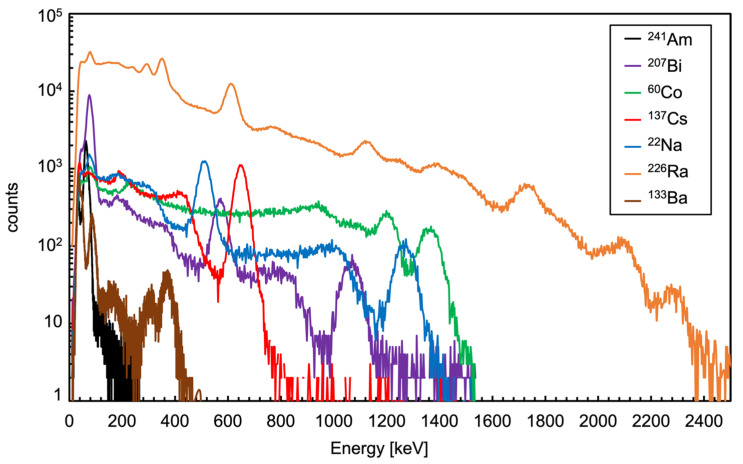
Spectra measured with the sources listed in Table 3. The vertical logarithmic scale was used in order to accommodate all the spectra in one single plot.

**Figure 28:**
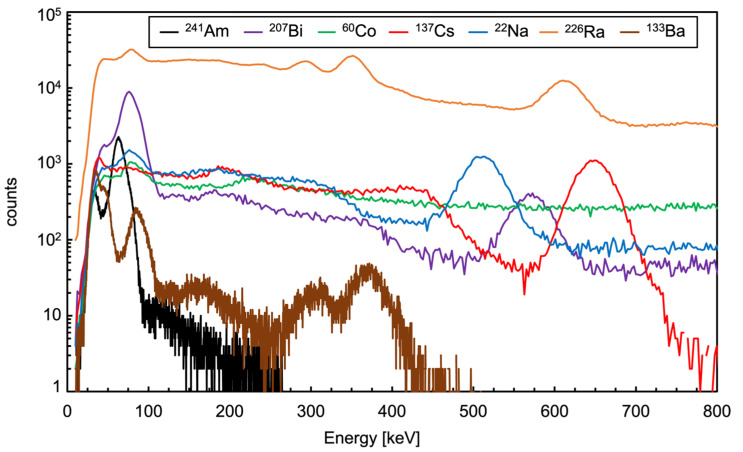
A zoomed version of Figure 27 expanding the 0 to 800 keV region.

The spectra measured with the sources listed in Table 3 are shown in Figure 27, the vertical logarithmic scale was used in order to accommodate all the spectra in one single plot. A zoomed version expanding the 0 to 800 keV region is shown in Figure 28. As a general indication useful for a comparison with other existing data on CsI(Tl), the measured FWHM resolution at 662 keV (i.e., ^137^Cs gamma ray) is 7.2%, well in line with values widely reported in literature, ranging from 5.6% to 10% [8,9,10,11,16,17]. Remarkably, such a resolution value, along with the full energy peak reconstruction up to and above 2000 keV, sounds quite encouraging in light of the tiny 1 cm^3^ crystal size and of the tinier 6 × 6 mm^2^ active area of the SiPM.

### 4.2. Test of Neutron Detection

**Figure 29:**
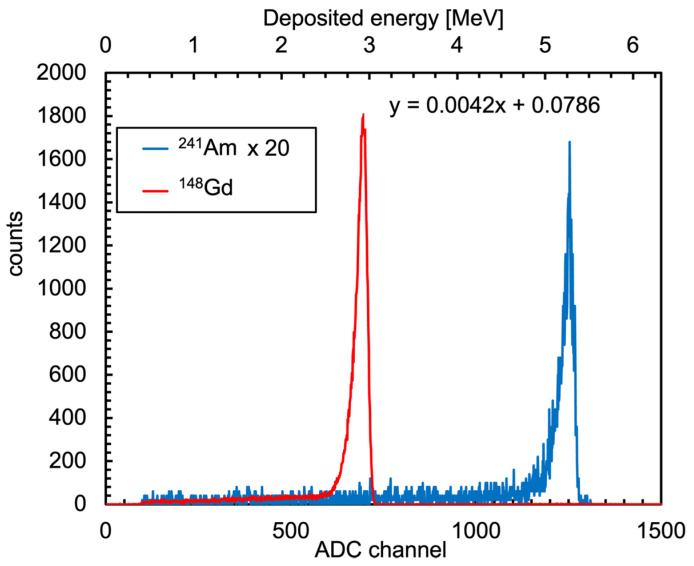
Spectra of the ^241^Am and ^148^Gd alpha sources and the channel-to-energy silicon diode calibration.

In order to calibrate a silicon detector in the required energy range, that is up to ≈5 MeV, two points are sufficient. A high-precision calibration is not required as the only needs are to check the triton spectrum endpoint around 2.7 MeV and to properly set the gamma discrimination threshold around 1.5 MeV. The silicon diode was connected to its front-end electronic board and exposed to an ^241^Am and to a ^148^Gd sources, which emit alpha particles of 5.486 and 3.183 MeV, respectively, at a distance of 1 mm. The energy loss of the alpha particles in the air and diode dead layers was taken into account. The resulting spectra and the channel-to-energy calibration are reported in Figure 29.

**Figure 30:**
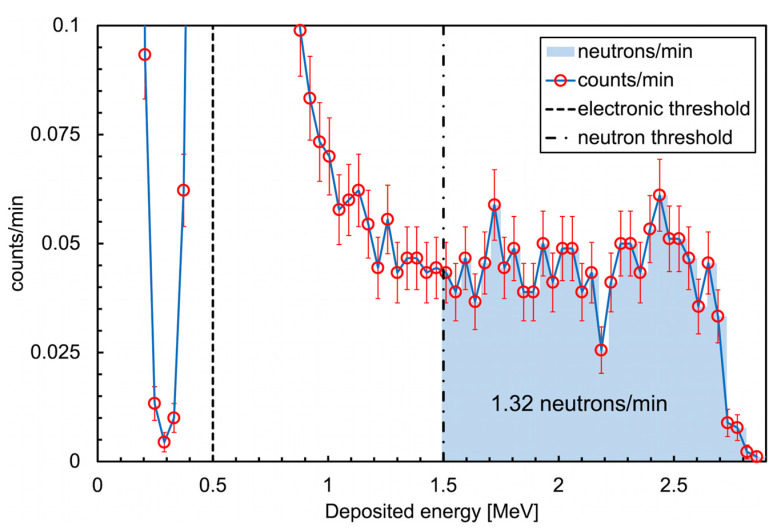
Deposited energy spectrum measured with the SiLiF detector with a low intensity AmBe neutron source.

Then, the ^6^LiF converter was assembled on top of the silicon diode and the detector was placed at about 10 cm from an AmBe neutron source and partially surrounded by a few plastic and paraffin blocks acting as moderator. Unfortunately, it was not possible to optimize this setup for logistic reasons. Moreover, the available source was low intensity as it only emits about 6000 neutrons/s in the whole solid angle; therefore, considering the 2.5% thermal detection efficiency, a solid angle fraction around 8 × 10^−4^ and a partial moderator geometry, the expected counting rate was of the order of 1–2 counts per minute. Nonetheless, a measurement of several hours produced the deposited energy spectrum shown in Figure 30, with a neutron counting rate well within the expectation. The corresponding spectrum taken without the ^6^LiF converter had no counts above 800 keV.

## 5. Conclusions

The test results of the MiniRadMeter gamma and neutron sensors indicate that it is suitable for the task it was designed to accomplish. Indeed, as shown in Section 2.1, the small CsI(Tl) crystal behaves well as a dosimeter which, in a few seconds, can provide a reasonable gamma dose rate measurement. Its additional spectroscopic features represent a useful tool for a preliminary indication of the typical emitting species possibly present in the environment. These results prove the complete feasibility of the 36 mm^2^ SiPM for the 100 mm^2^ crystal readout, in spite of its finite but large enough number of microcells (18,980) with fast recharge time (95 ns). The improvement of the measured energy resolution at 662 keV, from 9% reported in Section 2.1 to 7.2% in Section 4.1, is due to the additional reflective frame covering the black part around the sensitive area of the SiPM, that improves the light collection, and to the 20 µs integration time.

As for neutron detection, this is a more difficult task because a measurement of the same quality level as the gamma detection would require large and complex equipment that are definitely unsuitable for the UGV. Nonetheless, the device is capable of detecting the presence of a relevant neutron field in a matter of seconds by virtue of the superior gamma/neutron discrimination of the SiLiF technique, which is of the order of 10^−6^ or 10^−10^ when set to a 1 MeV or a 1.5 MeV threshold, respectively [36]. This means that, even in the case of a very low measured counting rate, down to 10^−2^ counts/s or lower, the signal can be reliably ascribed to neutrons [42] and will indicate their real presence in the environment, even though the quantitative dose rate information is poor due to the significant dependence of the detection efficiency on the energy shown in Section 2.2. 

The overall device weight is about 350 g; its maximum dimensions are currently 20 cm × 10 cm × 7 cm but could be reduced if necessary. The low weight and the compact size, along with the low cost, make it possible to update it into a deployable version. Indeed, a slim Li-ion battery could be easily incorporated inside the box, which would enable exploiting the onboard Wi-Fi interface for remote control and data transfer. This could be quite useful in the case of accident remediation as a number of MiniRadMeter sensors could be deployed by UGVs or flying drones on contaminated areas in order to map and monitor the radiation field. Another possible application is the use of the MiniRadMeter as a portable dosimeter, which was the original idea when it was initially devised, in which case the PET moderator would be removed and the human body itself would act as neutron moderator. In such a case, the Raspberry Pi4 microcomputer should be replaced by a more compact microcontroller and an FPGA unit, while the user interface software could be adapted and installed on a mobile phone. Indeed, the current configuration requires the presence of the operating system that limits the maximum counting rate to about 5 kHz, corresponding to 200 µSv/h for the gamma detector in spectroscopic mode and roughly to 16 mSv/h for 1 keV neutrons. If the gamma detector is operated in the counting mode, its limit is higher by one order of magnitude. The expected improvement of counting rate capability in spectroscopic mode, if employing a microcontroller to be programmed directly at a low level, is up to ≈25 kHz. 

The next step for the current version of MiniRadMeter will be the forthcoming test in a real environment that is planned as a final demonstration of the CLEANDEM project.

## Figures and Tables

**Table 1:** Main features of the CsI(Tl) scintillator material.

Property	Value
density	4.51 g/cm^3^
<Z>	54
attenuation coefficient at 662 keV	0.36 cm^−1^
light yield	60,000 photons/MeV
energy resolution @ 662 keV	5–10% FWHM
primary decay constant	960 ns
λ max emission	550 nm
refractive index @ emission max	1.79
cost of 1 × 1 × 1 cm^3^ crystal	≈EUR 30

**Table 2:** Possible candidate elements to be employed as converters for neutron detection.

Species	Cross Section [b]	Comment
^157^Gd	≈254,000	too many gammas (and toxic) [26,27]
^113^Cd	≈20,000	too many gammas (and toxic) [28,29]
^3^He	5333	expensive, being replaced in neutron detection applications, non-natural, produced in military reactors [30]
^10^B	3837	expensive, produces low energy alpha and ^7^Li particles but also gamma rays [31,32,33]
^6^Li	940	cheaper, easily deposited (^6^LiF), produces high-energy triton and alpha particles and no gamma rays [33,34]
^235^U	≈583	dangerous strategic material, radioactive, subject to radiation protection restrictions [33]

**Table 3:** Main energies of the gamma rays produced by the laboratory sources.

Source	Peak Energy [keV] Used for Calibration	Peak Energy [keV] Not Used for the Calibration Fit
^60^Co	1173, 1330	
^22^Na	511, 1274	170 *
^207^Bi	75, 570, 1064	
^137^Cs	662	184 *
^241^Am		59, 26
^226^Ra	295, 352, 609, 768, 1120, 1238, 1377, 1764	2204, 2248
^133^Ba		31, 35, 81, 356

* From Compton backscattering.

## Data Availability

The data presented in this study are available on request from the corresponding author.
